# Preamble Design and Collision Resolution in a Massive Access IoT System

**DOI:** 10.3390/s21010250

**Published:** 2021-01-02

**Authors:** Ailing Zhong, Zhidu Li, Ruyan Wang, Xingjie Li, Boren Guo

**Affiliations:** 1 School of Communication and Information Engineering, Chongqing University of Posts and Telecommunications, Chongqing 400065, China; lizd@cqupt.edu.cn (Z.L.); wangry@cqupt.edu.cn (R.W.); 2018210145@stu.cqupt.edu.cn (X.L.); 2 Key Laboratory of Optical Communication and Networks, Chongqing 400065, China; 3 Key Laboratory of Ubiquitous Sensing and Networking, Chongqing 400065, China; 4 School of Information Engineering and Communication, Beijing University of Posts and Telecommunications, Beijing 100876, China; plume@bupt.edu.cn

**Keywords:** random access, preamble collision resolution, timing advance capturing, Internet of Things

## Abstract

How to support massive access efficiently is one of the challenges in the future Internet of Things (IoT) systems. To address such challenge, this paper proposes an effective preamble collision resolution scheme to sustain massive random access (RA) for an IoT system. Specifically, a new sub-preamble structure is first proposed to reduce the preamble collision probability. To identify different devices that send the same preamble to the gNB on the same physical random access channel (PRACH), a multiple timing advance (TA) capturing scheme is then proposed. Thereafter, an RA scheme is designed to sustain massive access and the performance of the scheme is studied analytically. Finally, the effectiveness of the proposed RA scheme is validated by extensive simulation experiments in terms of preamble detection probability, preamble collision probability, RA success probability, resource efficiency and TA capturing.

## 1. Introduction

The rapid evolution of the wireless communication technologies are inviting all human beings to the era of the Internet of Everything [1]. The future IoT system is required to guarantee diverse requirements for various applications, such as huge bandwidth, low latency, high reliability, etc. [2,3]. As a result, huge amount of IoT devices need to access to the wireless networks to exchange data, which brings new challenge to the wireless network design [4]. Specifically, convention network scheduling mechanism is mainly designed for human to human communications that cannot sustain a large amount of simultaneous random access requests, since the preamble resources are quite limited. How to support massive random access for IoT devices consequently becomes a critical issue to realize the future Internet of Everything [5,6].

In general, existing research focuses on how to optimize the high-layer performance of an IoT system through resource allocations [7,8,9,10,11,12]. Such performance metrics include throughput, latency, access capacity, etc. The resource allocation schemes, however, can be realized only if the devices can be access to the IoT system. Hence, random access guarantee is one of the key issues to support massive devices to send data at the same time. Typically, more RA-attempting devices may lead to heavier load and more preamble collisions, which degrades the performance of an IoT system. Thus, it is necessary to design new preamble structure and RA scheme to reduce the congestion for the physical random access channel (PRACH). In the literature, the idea of performance improvement on PRACH can be generalized into three categories, i.e., resource increasing [13], user scheduling [14], and resource reusing [15]. In fact, resource increasing is costly and impractical, since the total resource of an IoT system is usually limited. In addition, preamble collision probability cannot be reduced through user scheduling since the number of preambles in an IoT system is finite. Differently, the idea of resource reusing can allow multiple devices to send identical preambles on the same PRACH, which is applicable to the massive access scenario. However, how to design a preamble structure and an RA scheme to efficiently reuse the PRACH resources for a massive access IoT system is still an open problem.

Motivated by this, this paper proposes an effective preamble collision resolution scheme to sustain massive random access for an IoT system. A new preamble structure consisting of the shared sub-preamble and the dedicated sub-preamble is first designed. Based on the new designed preamble, a TA capturing scheme is then proposed to identify multiple devices that send identical sub-preambles to the gBN on the same PRACH. Thereafter, we propose a random access scheme to reduce the preamble collision probability. Furthermore, we prove analytically that the proposed RA scheme can guarantee better performance than the conventional one proposed by the 3GPP. In addition, the effectiveness of the proposed scheme is validated by the extensive simulations. The contributions of this paper can be summarized as follows.

- A new preamble structure is designed, where preamble diversity and location diversity are both acquired. The designed preamble is able to guarantee a much lower preamble collision probability than the conventional preamble designed by 3GPP with ignorable detection impairment.
- A multiple TA capturing scheme is proposed based on the designed preamble. With the idea that the sub-preambles sent by a device suffer from the same channel environment, the TA value of each device can be captured, which can be further used to device identification.
- A new random access scheme is proposed and studied analytically. It is verified that the proposed scheme outperforms the conventional scheme in terms of preamble collision probability, RA success probability, etc. Hence, the proposed scheme is more applicable to the IoT systems where massive access is requested.

The remaining of this paper is organized as follows. Section 2 introduces the related works. Section 3 introduces new preamble sequence design. In Section 4, the preamble collision resolution scheme based on the proposed preamble structure is studied. In Section 5, the advantage of the proposed random access scheme is analyzed. Simulation results are presented and discussed in Section 6. Finally, the paper is concluded in Section 7.

## 2. Related Work

In the literature, performance improvement for massive access IoT systems is usually carried out from two aspects, i.e., cross-layer resource optimization and physical layer design.

On cross-layer resource optimization, researchers usually focused on performance optimization in terms of throughput, latency, and capacity. In [7], a throughput-oriented non-orthogonal random access (NORA) scheme was proposed to improve the access throughput for IoT networks by employing the technique of tagged preambles. With this, multiple devices shared the same physical uplink shared channel (PUSCH) for transmissions by multiplexing in the power domain. Wu et al. proposed a hybrid NORA and data transmission scheme to address the signalling overhead and resource allocation problems of IoT with non-orthogonal multiple access [8]. A relaying threshold-based random access and data transmission scheme for grouped IoT networks was proposed in [9]. Except for throughput optimization in [7,8,9], Chen et al. proposed an access class barring mechanism to accommodate heterogeneous IoT devices with different quality of service (QoS) requirements based on deep reinforcement learning algorithm in [10]. Additionally, a multi-slot pilot allocation scheme with unequal access latency protection for user equipments (UEs) was studied in [11]. The two solutions proposed in [10,11] devoted to reduce the latency in IoT networks. Moreover, in [12], the receiver is designed for the uplink transmission of a massive MIMO system to improve network access gain, where human-type communications and IoT communications coexisted.

On physical layer design, researchers usually focus on collision resolution. In [16], the multivariate Bernoulli model which exhibited the distributions of pilot states was proposed. With this, general correlation among device activities and relationship among the states of pilots assigned to one device could be specified. He et al. proposed a protocol which implemented multi-user detection and channel estimation by compressed sensing technology [17]. This protocol ensured that multiple collided packets could be recovered in one single time slot with low signaling overhead. The estimation of round-trip delays of multiple signals in NORA based on the maximum likelihood criterion was studied to assist the detection of collision in an IoT network [18]. Additionally, a contention control method that regulated the number of devices competing simultaneously for the RA channel was studied in a distributed manner [19]. Moreover, an RA scheme was proposed in [20], which introduced virtual preambles and associated them with RA channel indexes to discern multiple low-cost IoT devices. Works [16,17,18,19,20] all devoted to collision probability reduction. However, there was little attention on preamble collision in the physical layer of IoT. Please note that preamble detection is the prime of a successful random access.

Recently, preamble collision resolution was studied under the assumption that the TA values of the IoT devices were fixed [15]. Specifically, the TA value of each device was unique and the colliding devices could be discerned by the gNB. However, as the number of available preambles was finite, the scheme proposed in [15] was not applicable to the massive access scenario. Therefore, how to reduce preamble collision is still a challenge for an IoT system where huge amount of devices request access simultaneously, which motivates this paper.

## 3. Preamble Design

In this section, the conventional contention-based RA procedure and the structure of conventional preamble sequence are first introduced respectively. To address the drawback of conventional preamble sequence, a new sub-preamble sequence design is then proposed.

### 3.1. Conventional RA Procedure

**Figure 1:**
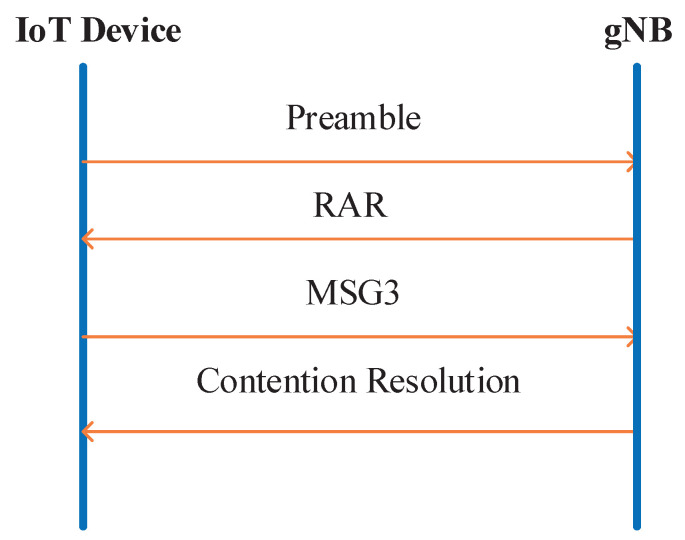
Conventional RA procedure.

The RA procedure includes two manners, i.e., contention-based manner and non-contention-based RA manner [21]. Since preamble collisions only occur in contention-based manner, this paper mainly focuses on contention-based manner. As depicted in Figure 1, a conventional RA procedure consists of four steps [22].

- Step-1: A device randomly selects a preamble from the available preamble sequence set, and then transmits the selected preamble on the available PRACH slot. If different devices transmit identical preambles on the same PRACH resources, RA contention will occur.
- Step-2: The gNB sends Random Access Response (RAR) message to the device after it detects a received preamble and captures the corresponding TA value. The RAR message contains the TA command, an uplink resource grant for MSG3 and a temporary cell-radio network temporary identifier (C-RNTI) for the device.
- Step-3: After receiving the RAR message, the device sends MSG3 to the gNB. MSG3 includes Radio Resource Control (RRC) connection request, scheduling request, and temporary C-RNTI. If preamble collision occurs, the gNB will be unable to decode the MSG3 data correctly.
- Step-4: If the gNB decodes the MSG3 successfully, it sends back the contention resolution message including RNTI acquired from the decoded MSG data. If a device receives its own RNTI within corresponding time window, RA procedure is accomplished successfully. Otherwise, the device restarts a new RA procedure.

Please note that in step-2, the gNB can only detect a specific preamble, but it cannot judge whether the detected preamble is sent by a single device or multiple devices. In addition, the preamble collisions cannot be detected before step-3. Once the collision occurs, it leads to a long RA delay and a waste of PUSCH resource.

### 3.2. Conventional Preamble

Zadoff-Chu (ZC) sequences are used as the root sequences of preambles due to its desirable periodic correlation. According to [23], a ZC root sequence with index *u* is defined as follows.

(1) $$x_{u}\left(n\right)=\exp\left[-j\frac{\pi un(n+1)}{N_{ZC}}\right],0\leq n\leq N_{ZC}-1,$$

where $N_{ZC}$ denotes the length of the ZC root sequence. For the *n*th root ZC sequence, the corresponding preamble with zero correlation zones of length $N_{CS}-1$ is defined by cyclic shifts

(2) $$x_{u,v}\left(n\right)=x_{u}\left(\left(n+C_{v}\right)\;\mathrm{mod}\;N_{ZC}\right),$$

where $N_{CS}$ and $C_{v}$ are given by [23]. A set of available preambles are generated from one or two root sequences by cyclic shifts.

Then, the received sequence in the gNB can be expressed by

(3) $$y\left(n\right)=\sum_{k=1}^{n_{k}}\sum_{e=1}^{n_{k,e}}h_{k,e}x_{k}\left[\left(n+t_{k,e}\right)\;\mathrm{mod}\;N_{ZC}\right]+z\left(n\right),$$

where $h_{k,e}$ denotes the *e*th multi-path channel coefficient for the *k*th device, $t_{k,e}$ denotes the delay of the *e*th multi-path for the *k*th device, $n_{k}$ denotes the total number of devices which attempt RAs on the same PRACH, $n_{k,e}$ denotes the amount of multi-path for the *k*th device, and z(n) denotes the circular symmetry complex Gaussian noise that can be expressed as

(4) $$z\left(n\right)=z_{r}\left(n\right)+jz_{i}\left(n\right),$$

where $z_{r}\left(n\right)$ and $z_{i}\left(n\right)$ both follow Gaussian distribution.

### 3.3. Sub-Preamble Sequence Design

**Figure 2:**
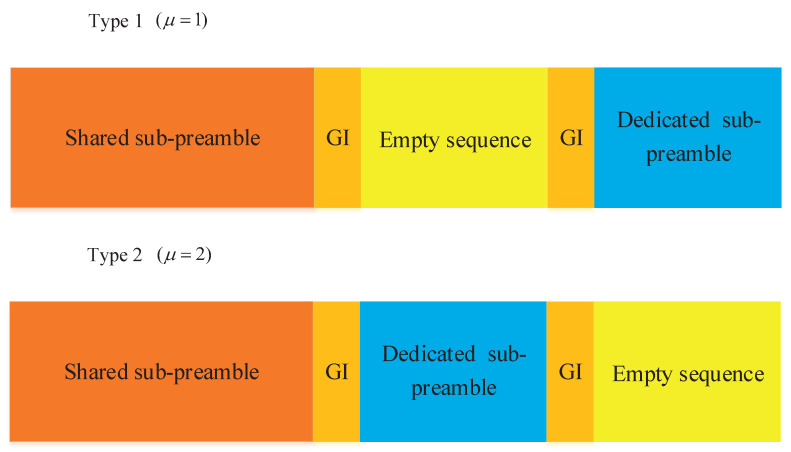
Preamble sequence design.

To address the drawback of the conventional preamble structure that easily leads to RA collision in a massive connective scenario, this paper proposes a new preamble structure with more diversities to reduce the RA collision probability. Specifically, the new preamble is consists of two sub-preambles and an empty sequence, as depicted in Figure 2. The sub-preambles are known as shared sub-preamble and dedicated sub-preamble respectively. The location of the shared sub-preamble is fixed. The location of the dedicated sub-preamble includes two types, which are denoted by μ∈{1,2}, as depicted in Figure 2. In addition, we use subscript i∈{s,d} to represent the variables corresponding to the shared sub-preamble and dedicated sub-preamble respectively.

The $n_{i}$th sub-preamble sequence with root index $u_{i}$ and cyclic shift index $v_{i}$ is generated as follows.

(5) $$x_{u_{i},v_{i}}\left(n_{i}\right)=x_{u_{i}}\left(\left(n_{i}+C_{v_{i}}\right)\;\mathrm{mod}\;N_{i}\right),0\leq n_{i}\leq N_{i}-1,$$

where $N_{i}$ denotes the length of the $n_{i}$th sub-preamble sequence, and $x_{u_{i}}$ denotes ZC root sequence that is obtained by Equation (2). As a result, the set of available sub-preamble can be obtained as

(6) $$S_{i}=\left\{x_{u_{i},v_{0}},x_{u_{i},1},...,x_{u_{i},N_{i}}\right\},i\in \left\{s,d\right\},$$

When an IoT device attempts to access to a gNB, it selects the shared sub-preamble and dedicated sub-preamble from the corresponding obtained set to compose a preamble randomly. In other words, each sub-preamble is generated by distinct ZC root sequence. According to the value of μ, the generated preamble sequence can finally generated by

(7) $$\begin{array}{cc} x_{u_{s},v_{s}}^{u_{d},v_{d}}\left(n\right) & =\alpha \left[x_{u_{s},v_{s}}\left(n_{s}\right),g_{1}\left(n_{g_{1}}\right),x_{0}\left(n_{0}\right),g_{2}\left(n_{g_{2}}\right),x_{u_{d},v_{d}}\left(n_{d}\right)\right],0\leq n_{i}\leq L_{i},0\leq n_{g_{i}}\leq L_{g}, \end{array}$$

or

(8) $$\begin{array}{cc} x_{u_{s},v_{s}}^{u_{d},v_{d}}\left(n\right) & =\alpha \left[x_{u_{s},v_{s}}\left(n_{s}\right),g_{1}\left(n_{g_{1}}\right),x_{u_{d},v_{d}}\left(n_{d}\right),g_{2}\left(n_{g_{2}}\right),x_{0}\left(n_{0}\right)\right],0\leq n_{i}\leq L_{i},0\leq n_{g_{i}}\leq L_{g}, \end{array}$$

where $x_{u_{s},v_{s}}\left(n_{s}\right)$ and $x_{u_{d},v_{d}}\left(n_{d}\right)$ represent the shared sub-preamble and dedicated sub-preamble respectively, $g_{m}\left(n_{g_{m}}\right)\;m\in \left\{1,2\right\}$ denotes Guard Interval (GI), $x_{0}\left(n_{0}\right)=0^{1\times L_{0}}$$\left(0\leq n_{0}\leq L_{0}\right)$ denotes an empty sequence with length $L_{0}$. Besides, $L_{i}$ denotes the length of the sub-preambles, $L_{g}$ denotes the length of the GI and α represents the transmission power. The preamble sequence generation scheme is summarized as Algorithm 1.

Without loss of reliability, generality and fairness, the following guidelines are considered: (1) The length of each sub-preamble sequence, i.e., $L_{i}$, should be a prime number to guarantee the periodic correlation of ZC root sequences. (2) The length of each sub-preamble should be selected as long as possible so that ZC root sequences can offer a good performance for TA capturing. (3) The length of the dedicated sub-preamble should be identical to the length of the empty sequence. (4) The total length of sub-preambles and GIs should be no longer than the bandwidth of PRACH that is equal to 839 or 139 according to different scenarios.
**Algorithm 1** Preamble generation scheme**Require:**     The sets of available sub-preambles $S_{s},S_{d}$; location index for the dedicated sub-preamble μ;**Ensure:**     A preamble sequence for an RA-attempting device $x_{u_{1},v_{s}}^{u_{2},v_{d}}\left(n\right)$;  1:  Randomly select a sub-preamble sequence from $S_{s}$ as a shared sub-preamble, $x_{u_{s},v_{s}}\left(n_{s}\right)$;  2:  Randomly select a sub-preamble sequence from $S_{d}$ as a dedicated sub-preamble, $x_{u_{d},v_{d}}\left(n_{d}\right)$;  3:  Randomly select a location index for the dedicated sub-preamble, μ;  4:  **if** μ=1 **then**
  5:    Generating a preamble according to Equation (7);  6:**else**  7:    Generating a preamble according to Equation (8);  8:**end if**

## 4. Preamble Collision Resolution Scheme

Based on our proposed preamble structure, this section studies how to reduce the preamble collision. The sub-preamble sequence reception scheme is first proposed. Then, we introduce how to capture multiple TA values in a single RA procedure. Thereafter, an integrated preamble collision resolution scheme is studied.

### 4.1. Sub-Preamble Sequence Reception

After sequence y(n) is received by the gNB, y(n) should be sliced into a shared sub-preamble and a dedicated sub-preamble. Since the sub-preamble structure is available to both IoT devices and gNB, the shared sub-preamble is apt to be obtained in the light of its predetermined length and location. Please note that the gNB is unable to recognize the dedicated sub-preamble, since the location index is random. However, the TA values obtained from the two types of the sub-preamble sequences sent by the same device should be identical, since those sub-preambles suffered from the same channel delay. Hence, for a specific shared sub-preamble, the corresponding dedicated sub-preamble can be identified via the TA values. Accordingly, the sub-preamble sequence reception scheme is summarized as Algorithm 2.

Please note that when multiple devices transmit their preambles on the same PRACH, $y_{s}$ consists of multiple shared sub-preamble sequences. Likewise, $y_{d}$ contain multiple dedicated sub-preamble sequences when the corresponding μ is configured identically. How to identify those overlapping sub-preamble sequences for different devices will be introduced in the next subsection.
**Algorithm 2** Preamble sequence reception scheme**Require:**    The received preamble sequence in the gNB y(n);  The length of the sub-preamble $L_{i}$;  The length of the GI $L_{g}$;  The length of the empty sequence $L_{0}$;  The sets of the local sub-preamble sequences $S_{i}$;**Ensure:**     The received shared sub-preamble sequence $y_{s}\left(n_{s}\right)$;   The received dedicated sub-preamble sequence $y_{d}\left(n_{d}\right)$; 1:  Intercept the first $L_{s}$ elements of y(n) as $y_{s}\left(n_{s}\right)$; 2:  Divide the residual sequence in the middle and obtain two sequences with the equal length $L_{d}+L_{g}$, i.e., ${\tilde{y}}_{d,candidate1}\left(n_{d}\right)$ and ${\tilde{y}}_{d,candidate2}\left(n_{d}\right)$; 3:  Remove the GI from ${\tilde{y}}_{d,candidate1}\left(n_{d}\right)$ to obtain $y_{d,candidate1}\left(n_{d}\right)$; 4:  Output $y_{d}\left(n_{d}\right)=y_{d,candidate1}\left(n_{d}\right)$ for μ=1; 5:  Remove the GI from ${\tilde{y}}_{d,candidate2}\left(n_{d}\right)$ to obtain $y_{d,candidate2}\left(n_{d}\right)$; 6:  Output $y_{d}\left(n_{d}\right)=y_{d,candidate2}\left(n_{d}\right)$ for μ=2;

### 4.2. Multiple TA Values Capturing Method

Based on the sub-preamble sequence reception, the gNB can correlate each sub-preamble with the local sequences respectively. The value of correlation calculation with a delay for the sub-preamble is expressed as

(9) $$\begin{array}{c} d_{i}\left(\tau_{i}\right)=\frac{1}{N_{ZC}}\sum_{n_{i}=0}^{L_{i}-1}{\left|y_{i}\left(n_{i}\right)\cdot c_{i}^{*}\left(\left(n_{i}-\tau_{i}\right)\;\mathrm{mod}\;L_{i}\right)\right|}^{2},\;0\leq n_{i}\leq L_{i}-1,\;i\in \left\{s,d\right\} \end{array}$$

where $\tau_{i}\in \left\{0,1,2,...,L_{i}-1\right\}$, $c_{i}\left(n_{i}\right)\in S_{i}$ denotes local sub-preamble sequence, and ${\left(\cdot \right)}^{*}$ denotes the complex conjugate. Additionally, the estimation TA value is related to the peak location of correlation, which can be expressed as

(10) $${\hat{\tau }}_{i}=\arg\max_{\tau_{i}}\left\{d_{i}\left(\tau_{i}\right)\geq r_{th}\right\},$$

where $r_{th}$ denotes the pre-determined threshold.

After the gNB detects the received shared sub-preambles, a set of TA values $\Gamma_{s}=\left\{\tau_{s}^{1},\tau_{s}^{2},...,\tau_{s}^{\theta }\right\}$ can be captured, where θ denotes the number of detected shared sub-preambles. Similarly, another set of TA values $\Gamma_{d}=\left\{\tau_{d}^{1},\tau_{d}^{2},...,\tau_{d}^{\sigma }\right\}$ can be obtained, where σ represents the number of detected dedicated sub-preambles. For ease of description, in the subsequence, the TA values in set $\Gamma_{s}$ are defined as shared TA values and those in set $\Gamma_{d}$ are defined as dedicated TA values. If $\tau_{s}=\tau_{d}$, the corresponding shared sub-preamble and dedicated sub-preamble are considered to belong to the same devices. This is because the shared sub-preamble and dedicated sub-preamble that sent by the same device always suffer from the same channel delay. Consequently, the gNB can identify preambles sent from different devices through comparing the shared TA value and the dedicated TA value.

**Figure 3:**
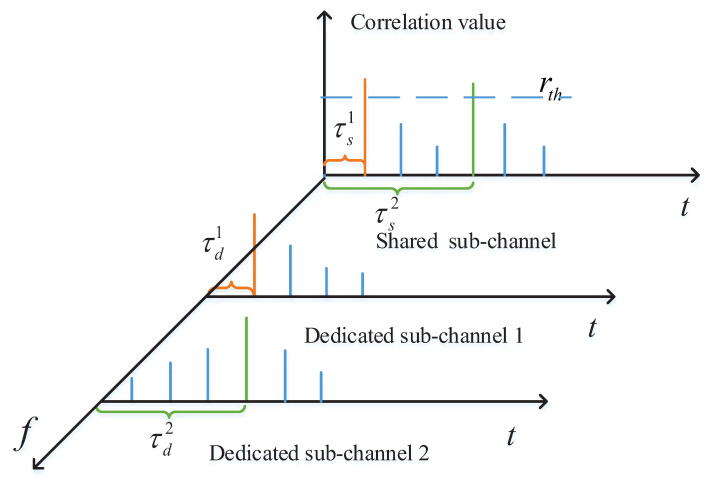
An example of capturing two TA values.

Figure 3 shows an example of capturing two TA values for two different devices. In the example, two RA-attempting devices send the shared sub-preambles with the same index $v_{s}$ and different dedicated sub-preambles on the same PRACH. Therefore, the gNB receives a overlapping preamble sequence. During the detection of the shared sub-preamble, two TA values can be captured by Equation (10) because of the same shared sub-preamble index. Please note that the gNB is unable to distinguish which TA value belongs to which device. However, during the detection of two dedicated sub-preambles, two TA values can be captured respectively. In detail, the TA values of the two shared sub-preambles $\tau_{s}^{1}$ and $\tau_{s}^{2}$ are captured on an identical detection zone, while the TA values of the two dedicated sub-preambles $\tau_{d}^{1}$ and $\tau_{d}^{2}$ are captured on two different detection zones. Hence, the gNB can identify a device by finding $\tau_{s}^{1}=\tau_{d}^{1}$ or $\tau_{s}^{2}=\tau_{d}^{2}$. The scheme of capturing multiple TA values is summarized as Algorithm 3.

Based on Algorithm 3, the TA value of each shared sub-preamble can be obtained by the gNB. If shared sub-preamble collision occurs, the TA value can still be captured with the corresponding dedicated sub-preamble. Only when the shared sub-preamble collision and dedicated sub-preamble collision occur simultaneously, the TA value is unable to be captured. Please note that sub-preamble collision implies more than one devices send dedicated sub-preambles with the same sub-preamble index on the same time-frequency resource. Hence, the probability that shared sub-preamble collision and dedicated sub-preamble collision occur simultaneously is low.
**Algorithm 3** Multiple TA value capturing scheme.**Require:**    Received shared sub-preamble sequence $y_{s}\left(n_{s}\right)$;  Received dedicated sub-preamble sequence $y_{d}\left(n_{d}\right)$;  The sets of the local sub-preamble sequences $S_{i}$;**Ensure:**    Captured TA value for the *k*th device $\tau^{k}$;  The shared sub-preamble index for the *k*th device $v_{s}^{k}$;  The dedicated sub-preamble index for the *k*th device $v_{d}^{k}$;  The location index for the *k*th device $\mu^{k}$;  1: **for all** $c_{s}\left(n_{s}\right)\in S_{s}$ **do**
  2:    Calculate $d_{s}\left(\tau_{s}\right)$ for the received shared sub-preamble $y_{s}\left(n_{s}\right)$ according to Equation (9);  3:    **if** $d_{s}\left(\tau_{s}\right)\geq r_{th,s}$ **then**
  4:         Record the sub-preamble index $v_{s}$ of $c_{s}\left(n_{s}\right)$ in $\Upsilon_{s}$;  5:         Calculate ${\hat{\tau }}_{s}^{1},...,{\hat{\tau }}_{s}^{\theta }$ according to Equation (10), and record them in $\Gamma_{s}$;  6:**    end if**  7: **end for**
  8: **for all** μ∈{1,2} **do**
  9:     **for all** $c_{d}\left(n_{d}\right)\in S_{d}$ **do**
 10:        Calculate $d_{d}\left(\tau_{d}\right)$ for the received dedicated sub-preamble $y_{d}\left(n_{d}\right)$ according to Equation (9); 11:        **if** $d_{d}\left(\tau_{d}\right)\geq r_{th,d}$ **then**
 12:             Record the sub-preamble index $v_{d}$ of $c_{d}\left(n_{d}\right)$ in $\Upsilon_{d}$; 13:             Calculate ${\hat{\tau }}_{d}^{1},\ldots ..,{\hat{\tau }}_{d}^{\sigma }$ according to Equation (10), and record them in $\Gamma_{d}$; 14:        **end if**
 15:    **end for**
 16: **end for**
 17: **for all** ${\hat{\tau }}_{s}^{k}\in \Gamma_{s}$ **do**
 18:     **for all** ${\hat{\tau }}_{d}^{k}\in \Gamma_{d}$ **do**
 19:          **if** ${\hat{\tau }}_{s}^{k}={\hat{\tau }}_{d}^{k}$ **then**
 20:               Output $\tau^{k}={\hat{\tau }}_{s}^{k}$ and the corresponding  $v_{s}^{k}$, $v_{d}^{k}$ and $\mu^{k}$; 21:          **end if**
 22:      **end for**
 23: **end for**


### 4.3. Random Access Scheme

Based on the multiple TA value capturing scheme, the random access scheme for multiple devices is revised as follows.

- Step-1: Sub-preambles selection and transmission. An IoT device randomly selects a shared sub-preamble and a dedicated sub-preamble from the available sub-preamble sets $S_{s}$ and $S_{d}$ respectively. Applying Algorithm 1, the device randomly selects a location index μ to generate a preamble sequence and then transmits it to the gNB on an available PRACH slot.
- Step-2: Random access response. When the gNB receives the preambles from multiple devices, applying Algorithm 2 to obtain the shared sub-preambles and dedicated sub-preambles respectively. Then, applying Algorithm 3, the gNB captures the TA value for each device and generates the corresponding RAR messages. The RAR message contains the TA command, uplink resource grant for MSG3, the shared sub-preamble index, the dedicated sub-preamble index and the location index μ.
- Step-3: MSG3 transmission. Devices can obtain their own RAR messages by confirming the shared sub-preamble index, the dedicated sub-preamble index and the location index μ. If RAR message is decoded correctly, the corresponding device will transmit MSG3 data on the PUSCH resource blocks that are allocated at step-2. As a result, the gNB can recognize that an RA collision occurs in this case.
- Step-4: Contention resolution. If the gNB successfully decodes MSG3, it will transmit a contention resolution message to the corresponding device. Such message contains the device ID that includes sub-preamble indexes and location index. Otherwise, nothing will be sent to the device. If the device receives contention resolution message, it will send back an ACK message and complete RA procedure. Otherwise, the device will restart a new RA procedure.

Under the proposed RA procedure, the preamble collision occur only if the shared and dedicated sub-preamble collisions occur at the same time. Additionally, the proposed RA scheme can still work when one type of the sub-preamble collision occur. In the next section, the effectiveness of the proposed RA scheme will be proved analytically.

## 5. Performance Analysis

In this section, the RA collision probability and the RA success probability under the conventional and proposed schemes are derived analytically.

### 5.1. Collision Probability

Under the proposed scheme, the preamble collision probability is associated with the probabilities of sub-preamble collisions as well as location collision of the dedicated sub-preambles. In other words, the preamble collision occurs when multiple devices select the same shared sub-preambles, the same dedicated sub-preambles as well as the same location indexes at the same time. Assuming that $n_{k}$ devices attempt RAs with $n_{p}$ available preambles. Consider a target device with deterministic preamble selection, the probability that another device users the same preamble holds as $1/n_{p}$. Since the devices are independent of each other, the preamble collision probability for the target device under the conventional RA [24] scheme holds as

(11) $$P_{collision}^{c}=1-{\left(1-\frac{1}{n_{p}}\right)}^{n_{k}-1}.$$

For the proposed RA scheme, we assume that $n_{k}$ devices attempt random access with $n_{p,s}$ shared sub-preambles and $n_{p,d}$ dedicated sub-preambles, and the number of available location index is $n_{\mu }$. For the target device and another device, if the shared sub-preamble, dedicated sub-preamble and location index used by those two devices are all the same, preamble collision will occur for the target device. The probability that another device uses identical shared sub-preamble, dedicated sub-preamble and location index at the same time holds as

(12) $$P_{\Delta }=\frac{1}{n_{p,s}}\cdot \frac{1}{n_{p,d}}\cdot \frac{1}{n_{\mu }}=\frac{1}{n_{p,s}n_{p,d}n_{\mu }}$$

Hence, the preamble collision probability for the target device under the proposed RA scheme holds as

(13) $$P_{collision}=1-{\left(1-P_{\Delta }\right)}^{n_{k}-1}=1-{\left(1-\frac{1}{n_{p,s}n_{p,d}n_{\mu }}\right)}^{n_{k}-1}$$

Comparing Equation (11) with Equation (13), when the number of available preambles in conventional RA scheme is equal to the number of available sub-preambles in the proposed RA scheme, i.e., $n_{p}=n_{p,s}$, $n_{p}=n_{p,d}$, the proposed scheme can guarantee lower collision probability than the conventional scheme.

### 5.2. RA Success Probability

During a complete RA procedure, the gNB allocates PUSCH resources for MSG3 transmission after successful preamble detection and TA capturing. For a conventional RA procedure, there are 4 cases for preamble collision and PUSCH resource allocation:

(1) $p_{1}^{c}=Pr\left\{\mathrm{preamble}\;\mathrm{collision},\mathrm{PUSCH}\;\mathrm{resource}\;\mathrm{allocated}\right\}$;
(2) $p_{2}^{c}=Pr\left\{\mathrm{preamble}\;\mathrm{collision},\mathrm{PUSCH}\;\mathrm{resource}\;\mathrm{unallocated}\right\}$;
(3) $p_{3}^{c}=Pr\left\{\mathrm{no}\;\mathrm{preamble}\;\mathrm{collision},\mathrm{PUSCH}\;\mathrm{resource}\;\mathrm{unallocated}\right\}$;
(4) $p_{4}^{c}=Pr\left\{\mathrm{no}\;\mathrm{preamble}\;\mathrm{collision},\mathrm{PUSCH}\;\mathrm{resource}\;\mathrm{allocated}\right\}$;

where $p_{4}^{c}$ represents the RA success probability in conventional scheme. Typically, the RA success probability in the conventional scheme is defined as the probability that a device uses an exclusive preamble and is allocated an exclusive PUSCH resource. Besides, $p_{1}^{c}$ represents the probability that the PUSCH resources are wasted. Moreover, the RA failure probability in conventional scheme is equal to $p_{1}^{c}+p_{2}^{c}+p_{3}^{c}$. Consequently, the RA success probability under conventional scheme can be expressed as

(14) $$\begin{array}{cc} P_{suc}^{c} & =p_{4}^{c}\\ \; & =\left(1-P_{collision}^{c}\right)\cdot P_{res}^{c}\\ \; & ={\left(1-\frac{1}{n_{p}}\right)}^{n_{k}-1}\cdot P_{res}^{c} \end{array}$$

where $P_{res}^{c}$ denotes the successful resource allocation rate for RA-step 3 under conventional scheme. Specifically, the PUSCH resources will be allocated to the IoT device in the RA-step 3 if its preamble is detected by the gNB sucessfully. Therefore, the successful resource allocation rate for RA-step 3 under the conventional scheme holds as

(15) $$P_{res}^{c}=\min\left\{1,\frac{n_{PUSCH}}{Pr\left\{\xi_{m}\geq 1\right\}\cdot \min\left\{n_{k},n_{p}\right\}}\right\},$$

where $n_{PUSCH}$ denotes the number of available PUSCH resources, $\xi_{m}$ denotes the number of devices selecting the *m*th preamble to perform access request. Since the devices are independent of each other, $\xi_{m}$ follows a Binomial distribution. Hence, we have

(16) Pr{ξm≥1}=1−Pr{ξm=0}=nk0(1−1np)nk=(1−1np)nk.

As a result, the RA success probability under conventional scheme can be obtained as

(17) $$\begin{array}{cc} P_{suc}^{c} & =p_{4}^{c}\\ \; & ={\left(1-\frac{1}{n_{p}}\right)}^{n_{k}-1}\cdot P_{res}^{c}\\ \; & ={\left(1-\frac{1}{n_{p}}\right)}^{n_{k}-1}\min\left\{1,\frac{n_{PUSCH}}{{\left(1-\frac{1}{n_{p}}\right)}^{n_{k}}\cdot \min\left\{n_{k},n_{p}\right\}}\right\} \end{array}$$

Similarly, there are 6 cases for PA collision and PUSCH resource allocation for the proposed RA procedure:

(1) $p_{1}=Pr${no shared preamble collision, PUSCH resource allocated};
(2) $p_{2}=Pr${no shared preamble collision, PUSCH resource unallocated};
(3) $p_{3}=Pr${shared preamble collision, no dedicated preamble collision, PUSCH resource allocated};
(4) $p_{4}=Pr${shared preamble collision, no dedicated preamble collision, PUSCH resource unallocated};
(5) $p_{5}=Pr${shared preamble collision, dedicated preamble collision, PUSCH resource allocated};
(6) $p_{6}=Pr${shared preamble collision, dedicated preamble collision, PUSCH resource unallocated}.

It is easily verified that the RA success probability under the proposed scheme is equal to $p_{1}+p_{3}$. On the contrary, the RA failure probability of the proposed scheme is equal to $p_{2}+p_{4}+p_{5}+p_{6}$. Besides, $p_{5}$ represents the PUSCH resources waste probability. Therefore, the RA success probability under the proposed scheme holds as

(18) $$\begin{array}{cc} P_{suc} & =p_{1}+p_{3}\\ \; & =\left(1-P_{collision}\right)\cdot P_{res}\\ \; & ={\left(1-\frac{1}{n_{p,s}n_{p,d}n_{\mu }}\right)}^{n_{k}-1}\cdot P_{res} \end{array}$$

where $P_{res}$ denotes the successful resource allocation rate under the proposed scheme. Additionally, let $\xi_{s,m}$ denote the number of devices selecting the *m*th shared sub-preamble, $\xi_{d,m}$ denote the number of devices selecting the *m*th dedicated sub-preamble, and $\zeta_{\mu }$ denote the number of devices selecting the μth index location. The successful resource allocation rate for RA-step 3 under the proposed scheme holds as

(19) $$\begin{array}{cc} \; & P_{res}=\min\left\{1,\frac{n_{PUSCH}}{Pr\left\{\xi_{s,m}\geq 1\right\}Pr\left\{\xi_{d,m}\geq 1\right\}Pr\left\{\zeta_{\mu }\geq 1\right\}\cdot \min\left\{n_{k},n_{p,s}n_{p,d}n_{\mu }\right\}}\right\} \end{array}$$

Here, as $\xi_{s,m}$, $\xi_{d,m}$, and $\zeta_{\mu }$ both follow Binomial distributions, there holds

(20) Pr{ξs,m≥1}=1−Pr{ξs,m=0}=nk0(1−1np,s)nk=(1−1np,s)nkPr{ξs,d≥1}=1−Pr{ξs,d=0}=nk0(1−1np,d)nk=(1−1np,d)nkPr{ζμ≥1}=1−Pr{ζμ=0}=nk0(1−1nμ)nk=(1−1nμ)nk

Thus, the RA success probability under under the proposed scheme holds as

(21) $$\begin{array}{cc} P_{suc} & =p_{1}+p_{3}\\ \; & ={\left(1-\frac{1}{n_{p,s}n_{p,d}n_{\mu }}\right)}^{n_{k}-1}\cdot P_{res}\\ \; & ={\left(1-\frac{1}{n_{p,s}n_{p,d}n_{\mu }}\right)}^{n_{k}-1}\min\left\{1,\frac{n_{PUSCH}}{{\left(1-\frac{1}{n_{p,s}}\right)}^{n_{k}}{\left(1-\frac{1}{n_{p,d}}\right)}^{n_{k}}{\left(1-\frac{1}{n_{\mu }}\right)}^{n_{k}}\cdot \min\left\{n_{k},n_{p,s}n_{p,d}n_{\mu }\right\}}\right\} \end{array}$$

## 6. Simulation Results

In this section, the performance of the proposed RA scheme is simulated and analyzed in terms of preamble detection probability, preamble collision probability, RA success probability, resource efficiency and TA capturing. The simulation parameters and their configurations are listed in Table 1. The simulation experiments are carried out on a MATLAB platform. In the simulation, preamble sequences are first generated for the conventional RA scheme and the proposed RA scheme respectively. Then different preambles with distinct TA values are transmitted to the gNB, suffering from a small-scale fading channel and additive white Gaussian noise. In convention RA scheme, the receive preamble sequence is detected by calculating the correlation value with all the local available preamble sequences. In the proposed RA scheme, the received sequence is divided into three sub-sequences, and each sub-sequence is detected by calculating the correlation values with all the corresponding available sub-preambles. The TA estimation is determined as the peak location of correlation. Once a TA is captured, the PUSCH resource block will be allocated to the user by the gNB in RA-step 3. An RA failure occurs when the TA cannot be captured or the PUSCH resource blocks are used up. To avoid the impact of randomness on the simulation results, we simulate each case for 10,000 times and output the mean results finally.

### 6.1. Preamble Detection Probability

To evaluate the impact of sub-preambles on the preamble detection probability, the proposed RA scheme is compared with conventional RA scheme when total *P* devices select exclusive sub-preambles to access to a gNB on the same PRACH. The preamble detection performance of the proposed RA scheme may be degraded, since the length of designed preambles is shorter than the conventional ones. To test the negative effect of sub-preambles, we randomly select sub-preamble sequence with different indexes from the available sub-preamble set.

**Figure 4:**
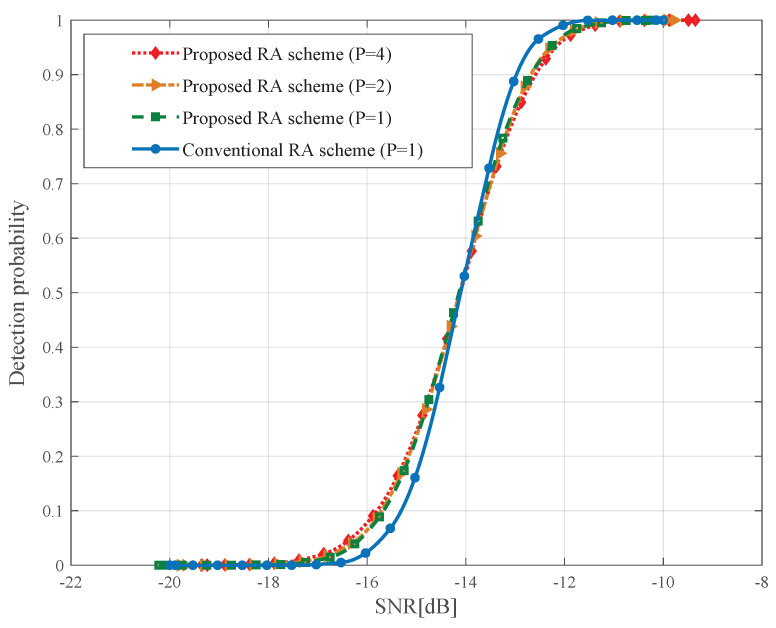
Comparison of the average preamble detection probabilities.

Figure 4 depicts the average preamble detection probability for different case of the number of devices *P*. The following observations are obtained: (1) When SNR is higher than −14 dB, the preamble detection performance of proposed scheme is a little poorer than conventional RA scheme; (2) The detection performance variation caused by varying amount of RA-attempting devices can be neglected; (3) The difference in the average preamble detection probabilities of proposed RA scheme and conventional RA scheme is negligible. The above observations imply that the sub-preamble structure does not impair the preamble detection performance. Hence, it is applicable to the IoT scenario where massive devices may request random access at the same time.

### 6.2. Comparison of Collision Probability

**Figure 5:**
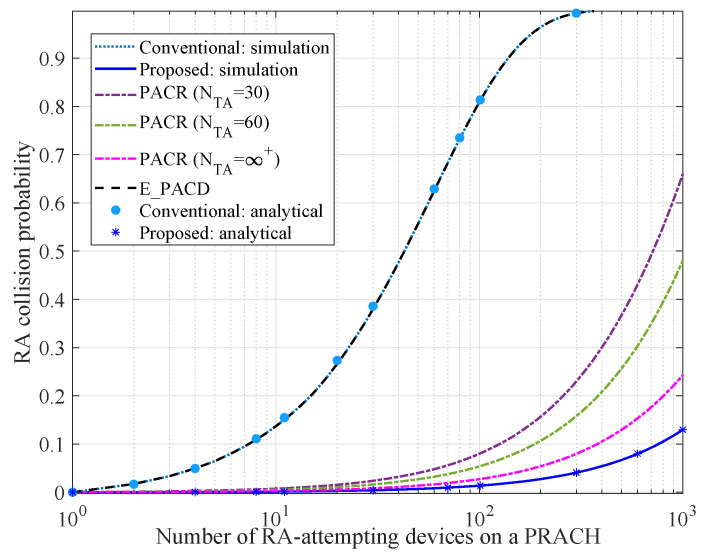
Comparison of collision probability.

Figure 5 depicts the relationship between the RA collision probability and the number of RA-attempting devices on a PRACH. In our simulations, the performance of the proposed RA scheme is compared with the conventional RA scheme, E_PACD scheme [14], and PACR scheme [15]. Firstly, the analytical results under the proposed RA scheme and that under the conventional scheme are both close to the simulation results, which verifies the accuracy of our analysis approach. It is also observed that the collision probability of the conventional RA scheme and that of E_PACD scheme are identical, since their collision probabilities only depend on the number of available preambles. Differently, the collision probability of PACR scheme depends on both the number of TA values $N_{TA}$ and the number of available preambles that can be allocated to the devices. Additionally, greater $N_{TA}$ means the gNB can detect more preambles on a PRACH, which can reduce the collision probability. However, $N_{TA}$ depends on the length of zero correlation zone of the detected preambles. Hence, $N_{TA}$ is finite. On the other hand, the RA collision probability of the proposed scheme depends on the size of sub-preamble sequence sets and the number of available locations of a dedicated sub-preamble. From Figure 5, we also observe that: (1) With the increase of the amount of RA-attempting devices, the collision probability of each scheme increases; (2) When the amount of RA-attempting devices is the same, the proposed scheme guarantees a lower RA collision probability than the other schemes, which means it is more suitable for massive connective scenarios.

### 6.3. Comparison of RA Success Probability

**Figure 6:**
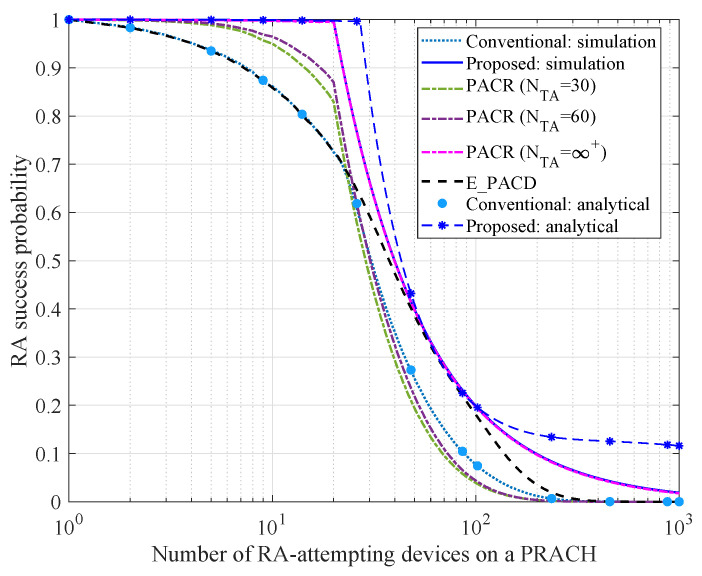
Comparison of RA success probability ($n_{PUSCH}=20$).

**Figure 7:**
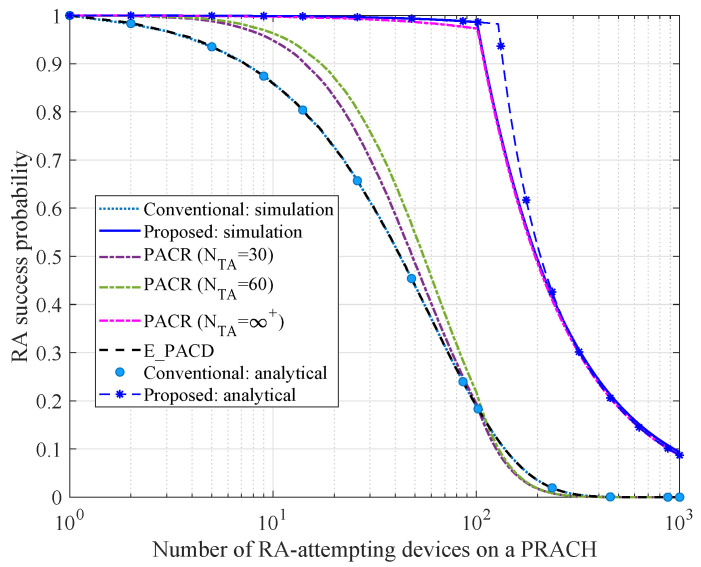
Comparison of RA success probability ($n_{PUSCH}=100$).

In this subsection, the RA success probability varying with the number of RA-attempting devices on a PRACH is analyzed. In our simulation experiment, we set $n_{p}=n_{p,s}=60$. Figure 6 and Figure 7 depict the simulation results with configuration $n_{PUSCH}=20$ and $n_{PUSCH}=100$ respectively.

Similar to Figure 5, the analytical results under the proposed and conventional schemes are both close to the simulation results, which validates the effectiveness of our theoretical analysis. As observed in Figure 6, the RA success probability under the proposed scheme is approximately 100%, when $n_{k}<20$. This is because the RA collision probability of the proposed scheme is close to zero. Therefore, all PUSCH resources can be allocated to the collision-free devices. When $n_{k}>20$, the RA success probabilities under the proposed scheme, the conventional scheme, PACR and E_PACR scheme all decrease rapidly, since the number of available PUSCH resources is insufficient. In this case, PUSCH resources shortage is the main influence on RA success probability. Besides, the RA success probability under the proposed scheme performs better than the other schemes. It is observed that the RA success probability under PACR scheme can be convergent to the proposed scheme only if $N_{TA}=\infty^{+}$ that is impractical in a real-world system. In Figure 7, $n_{PUSCH}$ is extended to 100. It is found that with the increase of $n_{PUSCH}$, the proposed scheme can guarantee a high RA success probability even though the number of RA-attempting devices on a PRACH beyond 100. Hence, it is verified that the proposed scheme can guarantee a higher RA success probability as long as the PUSCH resource is enough.

### 6.4. Comparison of Resource Efficiency

**Figure 8:**
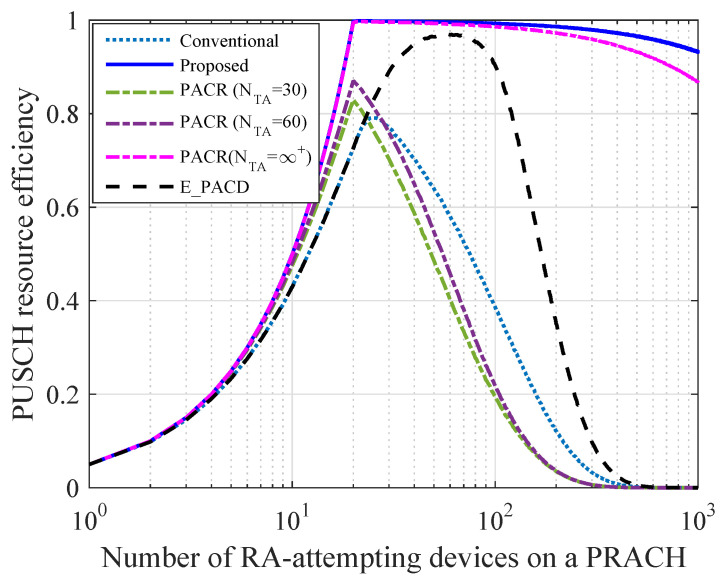
Comparison of PUSCH resource efficiency ($n_{PUSCH}=20$).

In this subsection, the resource efficiency is analyzed under different schemes. The resource efficiency is defined as follows

(22) $$\eta \overset{\Delta }{\;=}\frac{n_{success}}{n_{PUSCH}},$$

where $n_{success}$ denotes the number of PUSCH resources which are allocated to collision-free devices. As shown in Figure 8, the resource efficiency under the proposed scheme is higher than that under other schemes, especially when the amount of RA-attempting devices on the same PRACH is large. When $n_{k}>20$, the resource efficiency under the proposed scheme and that under PACR scheme both decrease with $n_{k}$. This is because the PUSCH resources are not enough to be allocated to the collision-free devices when $n_{k}>n_{PUSCH}$. However, the proposed scheme can still guarantee a high resource efficiency even though $n_{k}$ is large.

### 6.5. Analysis of TA Capturing Performance

In this subsection, the performance of TA capturing is evaluated in terms of TA capturing accuracy. Specifically, TA capturing accuracy is defined as

(23) $$\varepsilon_{TA}=N_{captured}/N_{preamble},$$

where $N_{captured}$ denotes the number of TA values which is captured correctly, and $N_{preamble}$ denotes the number of received preambles on the same PRACH resource. Compared with the conventional preamble, the sub-preamble is shorter. As a result, sub-preamble structure may degrade the performance of TA capturing. For the sake of performance analysis of the proposed multiple TA value capturing scheme, we focus on the collision of the shared sub-preambles. Assume that *M* preambles from *M* different devices are received on the same PRACH where the shared sub-preambles are set to the same index. Besides, the dedicated sub-preamble indexes and location indexes are randomly selected. The number of the available dedicated sub-preambles is set to 60, the number of available locations is set to 2.

**Figure 9:**
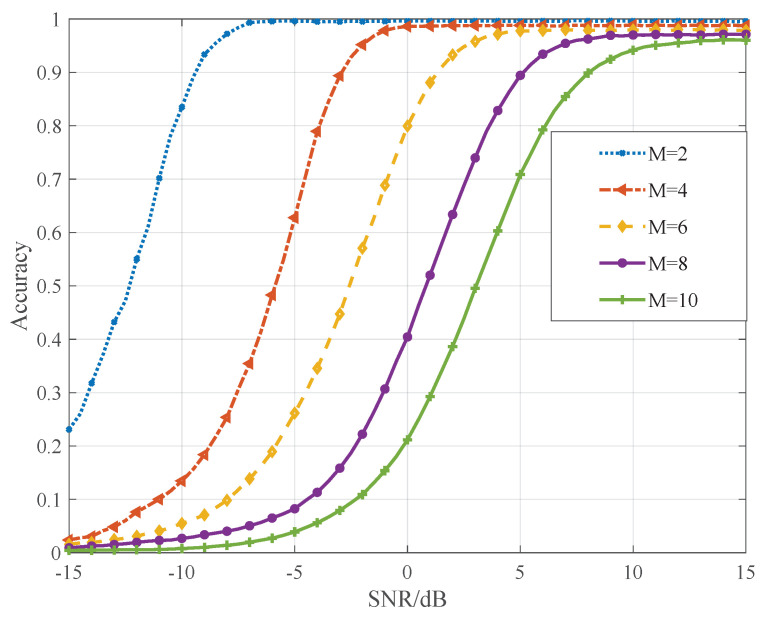
TA capturing accuracy.

As depicted in Figure 9, the number of preambles sent on the same PRACH is set to M={2,4,6,8,10}. It is observed that better TA capturing performance can be guaranteed when less devices transmit identical preamble on the same PRACH. For the case of M=2, the proposed scheme can guarantee over 80% accuracy even though the SNR is low (i.e., −10 dB). Similar accuracy can be obtained when SNR reaches 6 dB for the case of M=10 where interference is severe. Please note that the probability that 10 devices select the same shared sub-preamble is quite low. According to Equation (12), the probability is only 2.59% when 1000 devices request RAs with 60 available preambles.

## 7. Conclusions

In this work, a preamble collision resolution scheme was proposed for the IoT systems. The number of collision-free devices on the same PRACH was expanded by the proposed sub-preamble sequence structure. To obtain multiple TA values of the RA-attempting devices, a multiple TA capturing scheme was also proposed. Simulation results verified that the proposed RA scheme performed well in preamble collision probability, RA success probability, resource efficiency and TA capturing. In particular, the proposed scheme improved the RA success probability with ignorable degradation in preamble detection performance. Hence, the proposed scheme has a great potential for supporting massive access in the future IoT systems. In addition to the preamble design, preamble repetition is also an important way to improve the random access performance. Hence, our future work will focus on the preamble repetition mechanism design for a massive IoT system.

## Figures and Tables

**Table 1:** Simulation parameters.

Number of available shared sub-preambles	60
Number of available dedicated sub-preambles	60
Length of ZC sequence	839
Length of shared sub-preamble sequence	431
Length of dedicated sub-preamble sequence	199
Length of GI	5
Number of communication devices on a PRACH	1–1000
Signal strength (SNR)	−20∼15 dB

## Data Availability

The data presented in this study are available on request from the corresponding author. The data are not publicly available due to further study will be carried out.
